# Validation of a Tool-Based Visual Anorectal Examination Advanced Simulator for the Early Detection of Colorectal Cancer

**DOI:** 10.3390/jcm13051423

**Published:** 2024-02-29

**Authors:** Niamh Grayson, Reza Haghighi Osgouei, Renke Huang, Paris Tekkis, Fernando Bello, Christos Kontovounisios

**Affiliations:** 1 Imperial College Centre for Engagement and Simulation Science (ICCESS), London SW7 2AZ, UK; niamh.grayson20@imperial.ac.uk (N.G.); r.haghighi-osgouei@imperial.ac.uk (R.H.O.); renke.huang14@imperial.ac.uk (R.H.); f.bello@imperial.ac.uk (F.B.); 2 Department of Surgery and Cancer, Imperial College London, London SW7 2AZ, UK; p.tekkis@imperial.ac.uk; 3 Department of Colorectal Surgery, Chelsea and Westminster Hospital NHS Foundation Trust, London SW10 9NH, UK; 4 Department of Colorectal Surgery, Royal Marsden NHS Foundation Trust, London SW3 6JJ, UK

**Keywords:** simulation learning, proctoscopy, rigid sigmoidoscopy, rectal examination

## Abstract

Rectal examination through proctoscopy or rigid sigmoidoscopy is a common investigation in clinical practice. It is an important diagnostic tool for the workup and management of anorectal pathologies. Performing the examination can be daunting not only for patients but also for junior doctors. There are associated risks with the procedure, such as pain, diagnostic failure, and perforation of the bowel. Simulation-based training is recognised as an important adjunct in clinical education. It allows students and doctors to practice skills and techniques at their own pace in a risk-free environment. These skills can then be transferred to and developed further in clinical practice. There is extensive research published regarding the role of simulation-based training in endoscopy, however, we identified no published study regarding simulation-based training in rigid sigmoidoscopy or proctoscopy. This study aims to establish the initial face, content, and construct validity of a tool-based visual anorectal examination advanced simulator model for proctoscopy and rigid sigmoidoscopy. This innovative, highly realistic simulated environment aims to enhance the training of healthcare professionals and improve the efficiency of detecting and diagnosing distal colorectal disease.

## 1. Introduction

### 1.1. Background

Colorectal cancer is the fourth-most common cancer in the United Kingdom, with over 41,000 newly diagnosed cases of cancer each year [1]. Survival rates have increased to almost 60% in 5-year survival [2].

Colorectal adenocarcinoma typically develops from a polyp in the colon or rectum. This polyp progresses to an early adenoma, which becomes an advanced adenoma before becoming an invasive adenocarcinoma [3]. Hence, early diagnosis and management are imperative for a good prognosis and survival rates. Diagnostic tools include serum tumour markers, endoscopy, proctoscopy, sigmoidoscopy and colonoscopy, as well as radiological imaging, including computer tomography (CT) and magnetic resonance imaging (MRI) [1].

Proctoscopy and rigid sigmoidoscopy are simple, inexpensive bedside investigations commonly utilised in surgical clinics and operating theatres. They involve inserting the proctoscope or sigmoidoscope into the patient’s rectum and visualising the anus and rectum [4]. Unlike a colonoscopy, where bowel preparation is required, no bowel preparation is required, and there is no need for sedation or general anaesthesia. Rectal examination through proctoscopy and rigid sigmoidoscopy enables immediate diagnosis when a tumour is visualised, allowing for rapid investigation and management. It can also be used in localisation of rectal tumours and measurement from the anal verge, which is imperative in surgical planning [5]. Proctoscopy and rigid sigmoidoscopy have low complication rates, with rectal perforation occurring in 0.01% [6]. This is usually well tolerated by patients, with a low magnitude of pain reported [4]. However, the diagnostic value depends on the experience of the healthcare professionals and their exposure to rectal pathologies [3].

Simulation-based training is a well-established adjunct that supports acquiring and practising procedural skills. The American Board of Internal Medicine (ABIM) now recommends that residents initially train using simulation tools before attempting interventions or procedures on patients [7]. By simulating the experience, we can move away from the learning “on the job” style of traditional medical teaching. Trainees can learn and develop valuable skills and gain experience in a non-time-pressured, risk-free environment. This allows them to develop skill proficiency before applying it in the clinical setting [8]. Several computer-based simulators for endoscopy and colonoscopy exist in the market. The GI Mentor (Simbionix) [9], the ACCUtouch simulator (Immersion Medical) [10], and the EndoVR (CAE Healthcare) [11] are the most reported ones in the literature [12]. Each offers various simulation tasks with various scopes, from bronchoscopy to colonoscopy. To the best of our knowledge from reviewing the literature, this is the first proctoscopy and rigid sigmoidoscopy simulator system.

### 1.2. Literature Survey

The existing literature surrounding proctoscopy and sigmoidoscopy simulators and their use in training is very limited to non-existent. Sigmoidoscopy can be performed using a rigid or flexible tool. Flexible sigmoidoscopy is similar to colonoscopy in using a flexible, narrow tube with a light and tiny camera on one end. However, flexible sigmoidoscopy is limited to the rectum and the left colon, whereas colonoscopy explores the entire bowel.

An electronic search was performed on PUBMED, Google Scholar, and the Cochrane Library using the search terms “virtual reality” AND/OR “simulation” AND “sigmoidoscopy” AND/OR “proctoscopy”. No studies were identified reporting simulation training in proctoscopy. It was the same for rigid sigmoidoscopy. Given the scarcity of closely relevant previous work, we will briefly review the relevant studies on skill improvement and enhancement through simulation. Six studies were identified specifically investigating the role of simulation in flexible sigmoidoscopy training. Five studies investigated if training with the simulator improved students’ ability to perform sigmoidoscopy [13,14,15,16,17]. The last study by Datta et al. sought to validate PreOp flexible sigmoidoscopy as a training system [18].

#### 1.2.1. Endoscopy Simulation Training vs. No Training

In the study by Tuggy et al., [14] 10 residents with no previous endoscopy experience were randomly assigned to either the simulator or the control group. The control group had no previous sigmoidoscopy training before performing patient examination. The simulator group received 5 h of training on the Gastro-Sim flexible simulator [14].

The participants from each group then performed their first live flexible sigmoidoscopy on a patient, videotaped and scored based on timing, directional errors and percentage of colon visualised. Both groups then received 5 h of training on the Gastro-Sim simulator. This was followed by a repeat live flexible sigmoidoscopy with results collected under the same headings. This meant that the study had results from no simulator experience, 5 h of simulator experience, and 10 h of simulator experience [14]. As the time spent on simulation training increased, the time to 30 cm on scoping and the percentage of colonic mucosa visualised also increased. No difference was noted in pain scores or confidence levels predicted by patients across the two groups.

#### 1.2.2. Endoscopy Simulation Training vs. Conventional Patient-Based Training

Two studies compared virtual reality (VR)-based training to patient-based training [15,17]. Seldack et al. performed a similar trial comparing VR to patient-based training [17]. They compared the training group, which received 3 h of simulator-based training in addition to the standard training (1 week of patient-based flexible sigmoidoscopy training), to the control group, which completed the standard training. Patient discomfort scores were much lower in the group with simulator-based training when compared to the patient-based training group. There was no difference noted when comparing the skill scores. However, the simulator-based group did score higher in visualising the mucosa and identifying pathology.

Gerson et al. conducted their study differently [15], comparing exclusive VR-based and exclusively patient-based training. In this study, internal medicine residents were randomised into one of two groups, either training exclusively with a virtual reality simulator—Immersion Medical endoscopy simulator [10]—or exclusively bedside teaching. The residents in the simulator group had access to the machine with 6 sample cases and feedback on their performance for two weeks. The bedside teaching group received training on 10 patients under supervision and feedback from a consultant gastroenterologist. The results showed no difference between the groups regarding procedure time, patient satisfaction, or comfort scores. However, 72% of residents in the teaching group completed the scope independently, compared to 29% of the simulator group. Retroflexion was also completed by 84% of those trained bedside, compared to 56% of the simulator group [15].

#### 1.2.3. Endoscopy Training Implementation

The studies by Kneebone and Clark did not compare sigmoidoscopy training with standard training. Instead, they focused on the outcomes of implementing the training [13,16]. Kneebone et al. conducted a pilot study within a nurse practitioner endoscopy course. The course ran over 2 days and consisted of simulation-based scenarios with a flexible sigmoidoscopy simulator and individual tuition. Seven nurses participated over two days [13]. An objective increase in the percentage of mucosa seen on scoping—from 58.5% +/− 11.4% pre-VR training to 90.5% +/− 4.4%—was reported. This was in addition to an improvement in the efficiency of the performance of sigmoidoscopy [13].

Clark et al. introduced the GI mentor to their training programme for 13 surgical residents. During the GI training programme, there were monthly assignments of 10 simulator scenarios covering various endoscopies—OGD, colonoscopy, and flexible sigmoidoscopy [16]. They used the GI Mentor simulator [9], collecting data over two years from 13 surgical trainees and 5 junior and 3 senior surgical residents, who completed the assigned cases on the simulator. Performance efficacy was measured by the total time for examination by the percentage of mucosa visualised. Both groups’ performance efficacies increased throughout the study period [16].

#### 1.2.4. Validation of Simulator Training

Datta et al. [18] conducted a study to validate a flexible sigmoidoscopy simulator training system. Their study divided participants into three groups: novice—no scoping experience, intermediate—5 to 50 lower GI endoscopies performed, and expert—over 200 scopes performed independently. They reported a significant difference in the three groups regarding the percentage of colonic mucosa visualised, with the novice group having the lowest and the expert group having the highest. They concluded that the system proved valid as a training tool [18].

Overall, the literature reviewed is positive regarding simulator training in sigmoidoscopy.

## 2. Materials and Methods

### 2.1. System Description

**Figure 1:**
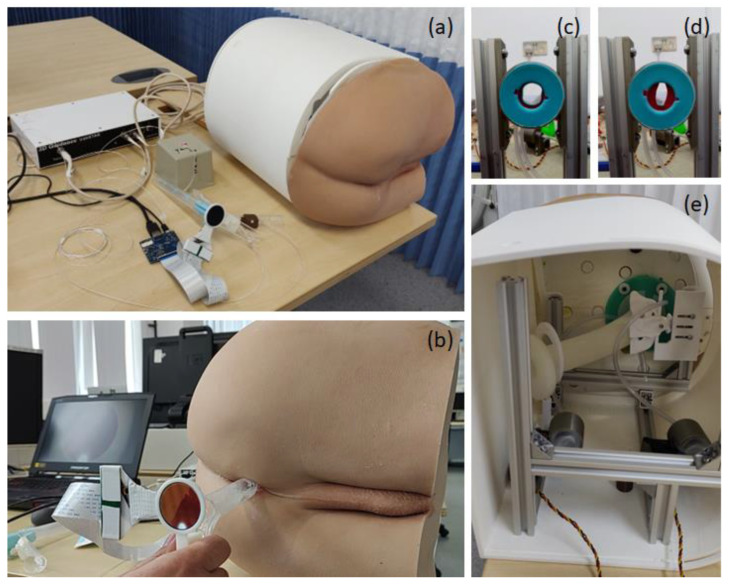
(**a**–**c**) Tool-based visual anorectal examination simulator. (**b**) A proctoscope mounted with a round LCD to visualize virtual conditions. (**c**,**d**) A pneumatic sphincter actuator (**e**) integrated into the simulator.

We developed a training system for practicing tool-based visual anorectal examination using a disposable proctoscope and rigid sigmoidoscope. The system consists of both hardware and software components. All hardware components are enclosed in a 3D-printed casing to form a compact benchtop model (Figure 1).

Twelve different cancer and non-cancer conditions (Table 1) were implemented in the software program and rendered onto a round liquid-centred display (LCD) mounted on the back of the tools. The system also includes a pneumatic sphincter actuator to render various anal tones, Figure 1c,d. During the examination, the user inserts the tool into the prosthetic buttock and scopes the anus, rectum and distal colon. Looking at the display mounted on the tools for any suspicious signs, just as they would check through the eye of the scope in a real-life scenario.

The hardware includes a realistic prosthetic buttocks model, a pneumatic sphincter actuator, a 3D-printed coccyx bone, and a silicone rectum. These components are mounted on an aluminium frame and covered with a 3D-printed casing (Figure 1).

We used the same examination tools, proctoscope or rigid sigmoidoscope, as in the colorectal surgery clinic. An electromagnetic tracking sensor system (3D Guidance trakSTAR) [19] was mounted to the instrument handle to track the 3D position and orientation of the tool during the examination. In addition, a small round LCD was mounted on the back of the tool above the handle to serve as a visor for visualisation of the virtual conditions. Proper mounting adapters to attach the sensor and LCD to the instrument were designed and 3D printed. The sensor was connected to a laptop via a USB connection and the LCD via an HDMI connection. As the participant advanced and navigated the instrument, the display on the screen changed appropriately, simulating real-life proctoscopy/rigid sigmoidoscopy.

In addition, the rigid sigmoidoscope includes a hand pump to introduce air into the rectum to expand it, exactly as is performed in clinical practice. The pneumatic sphincter actuator consists of two air chambers mimicking human anal sphincter muscles. Each of the chambers is driven by a bellows that is pressed by a servo motor to render one of four predefined anal pressures. Both servo motors are controlled using an Arduino module [20,21].

**Figure 2:**
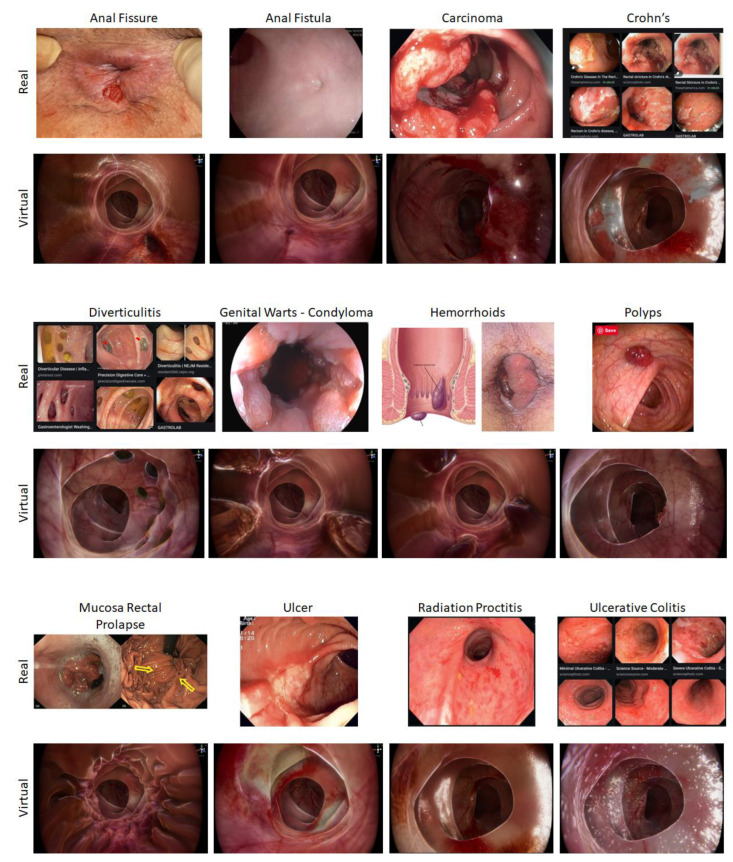
Various modelled conditions.

The twelve different conditions created for the software (Table 1) were designed to look like real cases (Figure 2). A wide range of pathologies were selected, covering cancer and benign conditions. The conditions included were those which are commonly encountered in standard clinical practice. RC02, RC03, RC07, RC08, and RC12 were all programmed for the proctoscope as they are all more distal pathologies and therefore visualised with this tool. The remaining pathologies were created for the rigid sigmoidoscopy tool. The virtual pathologies were created from sample images of real pathologies using ZBrush (2021.6, Pixologic, Schererville, IN, USA) and Unity 3D (2020.3.48, Unity Technologies, San Francisco, CA, USA) [22,23]. A senior colorectal consultant surgeon (CK) discussed, reviewed, and approved the cases and pathology software programs.

The simulator software reads the position and orientation of the real tool via the electromagnetic tracking sensor to move and rotate the virtual counterpart. To increase the realism of the interaction, the simulation includes soft tissue deformation whereby the colorectal tissue visualised moves in response to its interaction with the instrument. The virtual rectum is responsive to the virtual tool movement and rotation, opening with tool insertion and collapsing with tool withdrawal.

### 2.2. Methodology

We conducted the experimental study in the Imperial College Centre for Engagement and Simulation Science (ICCESS) at Chelsea and Westminster Hospital. Participants were recruited on a volunteer basis.

Ethical approval was sought from an application to the Education Ethics Review Process team at Imperial College London. The study was deemed very low risk with no potential to harm or result in negative consequences. Ethical approval was granted on 17 June 2022 (Ref. No. EERP1920-102). 

Participants were first asked to complete a pre-study questionnaire. This questionnaire provided information on their clinical experience years, specialist colonoscopy skill level, and previous simulators and proctosigmoidoscopy experience. The intention was to collect background information about the participants, including their relevant practice, level of training and use of simulators, and their perception of the challenges in performing tool-based rectal examination. They answered four questions regarding the ease and challenges of performing a rectal examination with proctoscopes and rigid sigmoidoscopes. These were scored on a Likert scale of 1–5, with 1 being very easy and 5 being very difficult. The questions included inserting the tools, navigating the tools, reaching anatomy, and identifying pathology.

After completing the questionnaire, the participants were shown the simulator and benchtop model. They were asked to perform five separate procedures on the benchtop model: one healthy condition using a proctoscope, two conditions using a proctoscope, and two conditions using a rigid sigmoidoscope. Gloves and lubricants were provided to simulate the clinical setting.

For each condition, they identified and assessed the colorectal mucosa. Whilst performing the examination, they were given no feedback or information that may have assisted their diagnosis. 

Following completion, the participants answered the second part of the questionnaire. Firstly, they answered if the condition was normal or abnormal (i.e., pathological). In this part, they answered with a diagnosis selected from a list for each condition (Table 1). Once they had completed this, they were informed of the correct condition and were then asked to rank the similarity of the condition on a scale of 1 to 5, with 1 being not similar at all and 5 being very similar.

At the end of their session, participants completed a post-study questionnaire also using a Likert scale, scoring 1–5 for each answer (Table 2), with a free-text area at the bottom for them to include any further feedback or suggestions.

## 3. Results

### 3.1. Participants Background and Experience

As per the study’s inclusion criteria, we initially aimed to recruit 10 consultant colorectal surgeons and 10 colorectal surgical trainees, covering a range of experience in examining colorectal patients, specifically with proctoscopy and rigid sigmoidoscopy. However, due to pressures on the service and availability, we could recruit only seven consultants, eight surgical trainees, and two specialist registrars in surgery (SpR) (Table 3). We intended to gain feedback from experienced participants on the fidelity and adequacy of the simulator as a training resource, and feedback on the usability and adaptability of the prototype from less experienced participants. The consultant participants have been trained and assessed on procto-sigmoidoscopy examinations, performing them daily. The surgical trainees and SpRs had different experience levels, from pure observation to independently performing them in a clinical setting.

Of the 17 participants, 9 had previous simulator training exposure. This exposure included resus simulation, laparoscopic simulation, prostate stimulation, and trauma simulation, as well as training with a hip intra-muscular nail simulator, chest drain simulator, lumbar puncture simulator, and knee aspiration simulator. Laparoscopic simulator training was the most commonly occurring previous training tool with three participants reporting having trained on it.

Of the 17 participants, 8 reported previous simulation training experience, compared to only 1 of the 7 consultants in the study. This may suggest that simulation training is becoming more common within medical training. Although 41% (7/17) of participants had previous VR/simulation training exposure, none of this was in rectal examination.

### 3.2. Pre-Study Questionnaire

**Figure 3:**
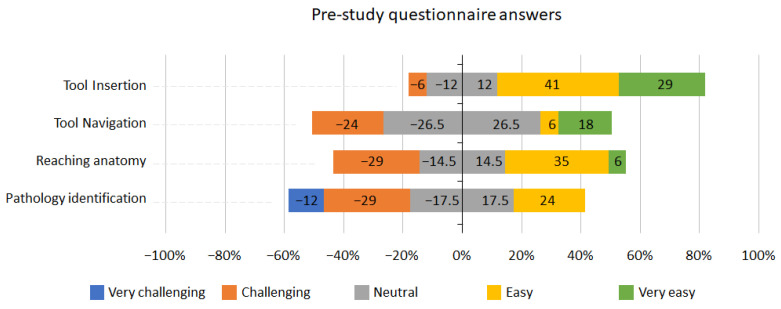
Pre-study questionnaire answers.

Figure 3 depicts the pre-study questionnaire results of the seventeen participants. 29% of participants answered that they found navigating the tool challenging, 29% found reaching anatomy challenging, and 41% found identifying pathology challenging or very challenging. 

Twelve of the seventeen participants responded to the question on how the training of rectal examination can be improved. The answers are listed in Table 4 below.

### 3.3. Assessment of Condition

The most clinically common and relevant conditions were selected from the list (Table 1). These included the normal scope, the anal fissure, the colonic carcinoma, diverticulitis, and haemorrhoids. 94% of the participants (16 out of 17) answered correctly when asked to identify normal mucosa on scoping the VR normal simulator.

Identification of haemorrhoids was the most successfully diagnosed pathology on scopes. 94% (16/17) identified this correctly, followed by anal fistula in 59% (10/17). Carcinoma and diverticulitis were identified correctly with 24% (4/17) of the participants. 

**Figure 4:**
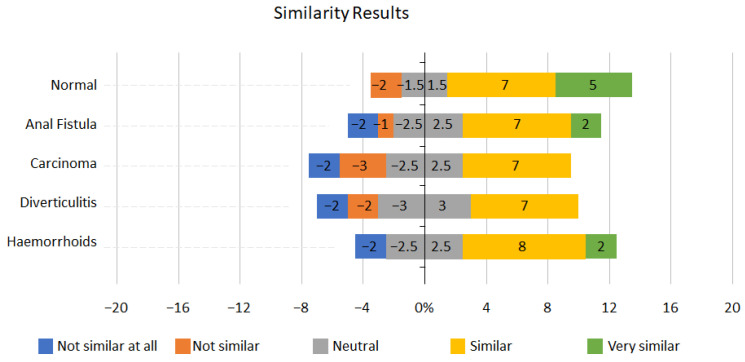
Similarity results.

For each condition on the scope, the candidates ranked the similarity from 1 to 5, with 1: not similar at all and 5: very similar. The results can be seen in Figure 4 below.

Normal mucosa scored very highly on the similarity measure, with 70% (12/17) of participants recording it as similar to real-life scopes. Haemorrhoids also scored well, with 59% (10/17) of candidates scoring either similar or very similar. Over half of the participants, 53% (9/17), reported the anal fistula simulation as similar to real-life conditions. 

However, when considering these results, it is important to consider participants’ experience. Without previous relevant rectal examination experience and exposure to the examined pathologies, their judgement of similarity to the real-life pathology may likely be inaccurate. 

The similarity scoring was challenging to interpret. Initially, this may be due to the range of experience within the group. Except for the normal mucosa being reported as similar by 70% (12/17) of participants, no consensus was reached for the other conditions. 

This may be due to a variety of reasons. Firstly, we did not ask if the participant had encountered the pathologies in real life. If they have no previous real life scoping exposure to such a condition, then the similarity score they award the condition may be regarded as invalid. Secondly, the data set is small; once we excluded the 5 more junior participants, it left us with only 12 participants (7 consultants and 5 higher surgical trainees) to analyse the data. Third, not all participants were presented with all the conditions, just a selection of five conditions. With a larger data set (increased number of participants and increased exposure to all the conditions), trends would begin emerging, and similarity to the condition could be noted.

### 3.4. Post-Study Questionnaire Results

**Figure 5:**
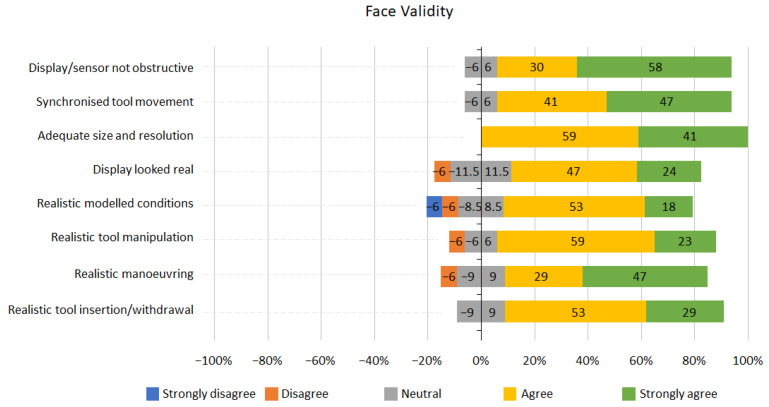
Face validity Likert scale results.

**Figure 6:**
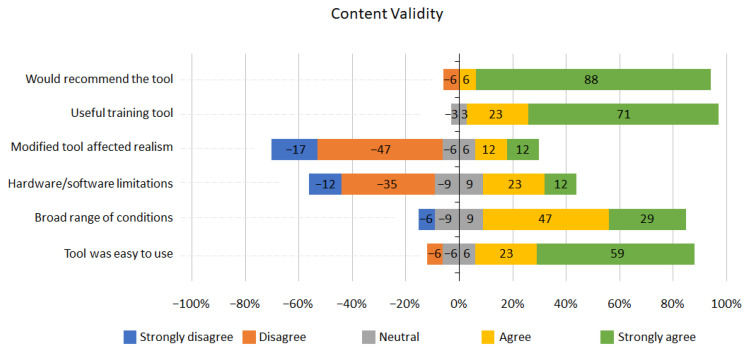
Content validity Likert scale results.

Graphs representing the results for face and content validity are shown below (Figure 5 and Figure 6). Overall, the results from the face validity part of the questionnaire were positive (Figure 5); 82% of participants agreed or strongly agreed that the tool insertion and withdrawal was realistic or very realistic; 76% of participants reported the round display looked real. For all face validity questions, participants were either neutral or agreed that the tool had good face validity, except for the 12% of participants who disagreed that the modelled conditions were visually realistic.

The feedback regarding content validity was overwhelmingly positive (Figure 6). However, 6% of participants reported that the range of conditions were not broad enough; 35% of participants reported that the simulator had some hardware and software limitations as a training tool, and 24% felt the realism of the tool was affected by modifications. Nevertheless, overall, 94% percent of participants agreed that the tool was useful as a training tool and that they would recommend the training tool.

## 4. Discussion

This study aimed to investigate if the benchtop model and virtual proctoscopy, and rigid sigmoidoscopy simulator would prove valid in their use as training tools. From our pre-study questionnaire, we identified that there is a clear gap in training medical students and doctors in rectal examination. The use of simulation is becoming more popular in medical training. From our literature review, this is the first visual anorectal examination simulator. 

The validity of the proposed visual anorectal examination simulator was established through multiple criteria.

### 4.1. Fidelity

1. Physical fidelity—The simulator is of high physical fidelity. The tools used were taken from the clinical setting, therefore identical to real clinical practice. The buttocks were created like a human buttock through a combination of silicon casting and 3D printing moulding. In the post examination questionnaire, none of the participants disagreed with the tool being realistic.
2. Psychological fidelity—The cognitive demands of performing a proctoscopy/rigid sigmoidoscopy in looking for pathology and identifying it were replicated in this study, but the additional cognitive demands of performing a rectal examination in real life, such as managing a real-life patient, were not addressed. This could be improved by including clinical scenarios in the simulation, perhaps with the addition of simulated patients, as one participant mentioned: “Improve feedback of patient discomfort to make it more realistic”.
3. Affective fidelity—Given that the principal goal of this simulator was to improve the participants’ ability and comfort level in managing the instruments and identifying pathology, affective fidelity was also not addressed in this study but, once again, this could be achieved with the addition of simulated patients to the simulation.
4. Ergonomic fidelity—Ergonomic fidelity was achieved through the anatomically accurate rectal model that provided haptic feedback recreated from real rectal examinations. The simulator’s built-in software reads the position and orientation of the tool, updating the virtual image appropriately based on the manipulation of the physical instruments. Soft tissue deformation enabled the virtually rendered tissues to respond to the insertion of the instrument.

### 4.2. Face and Content Validity

Face validity was assessed from the questionnaires completed by the participants at the end of the study. The results shown in Figure 5 indicated that the simulator has a high face validity, with most participants (76%) agreeing that the tool and its operation were realistic; 100% of participants answered that the size and resolution of the images displayed were adequate; 71% agreed that the tool allowed for realistic visual observation.

User experience and content validity scored highly in the questionnaires (Figure 6). 94% of participants agreed it was a useful learning tool and would recommend it; 64% of participants declared that tool modification unaffected simulation realism, and 47% did not report hardware/software limitations. In addition, 76% of participants assumed the range of conditions was broad enough, and 82% reported that the tools were easy to use. We have identified a few areas to improve the usability of the system—for example, improved software in terms of rendering quality and responsiveness to the user actions to increase the simulation realism. In addition, using a wireless tracking sensor and a less bulky round display adaptor could notably reduce the hardware limitations and improve the stability and portability of the system. Adding more conditions, along with simulating various stages of cancer conditions, could reduce software limitations and enhance the range of available conditions.

### 4.3. Construct Validity

It is difficult to prove the construct validity of a simulation tool, particularly in the single session this study conducted and with limited numbers of participants across the range of experience.

Regarding normal and abnormal mucosa, 94% (16/17) of participants correctly identified normal mucosa on the scope. This is important as it concords with a valid simulation of normal mucosa on scoping.

As previously discussed, the scoring of the correct answers may have been affected by the participants in the study. Seven participants were surgical consultants, with the remaining 10 ranging from core surgical trainees to specialist surgical registrars. Further work is needed to establish construct validity properly.

### 4.4. Pedagogical Validity

Given that this study’s focus was to assess its initial validity as a training tool, pedagogical validity was not considered. Such validity needs to be considered before implementing the tool into a curriculum. For example, it would be necessary to establish how many sessions are required and what are the specific learning outcomes of the programme, such as correctly manipulating the instruments, correctly identifying all pathologies, etc. A teaching session before participation for trainees to learn the theory surrounding the examination, its importance, risks, and associated difficulties could also be included. It would also be important to provide trainee feedback and answer any questions they may have during the sessions.

## 5. Conclusions

In the UK, the NHS long term plan aims to improve early detection and diagnosis (ED&D) of colorectal cancer from 54% in 2017 to 75% in 2028. Currently, only about 25% of colorectal cancer conditions are diagnosed in stages 1 and 2 from GP referrals. Quick screening procedures, such as tool-based rectal examination using a proctoscope and rigid sigmoidoscope, can be performed in GP surgeries and community clinics to improve ED&D. If performed adequately and in time, proctoscopy and rigid sigmoidoscopy can improve ED&D saving lives and reducing treatments costs drastically. However, such examinations are not done often enough because doctors do not possess adequate manipulative and diagnostic skills in using tools and interpreting visual observations. This results in a lack of confidence, reducing the number of examinations conducted.

In line with the NHS long-term plan, we have developed a realistic training system to enable clinicians to practice tool-based rectal examination using a real proctoscope or rigid sigmoidoscope to improve their skills and confidence. The system enables using real tools, a proctoscope and rigid sigmoidoscope, on a realistic prosthetic buttock, rendering 12 different cancer and benign colorectal conditions on a small round display attached to the tools. We evaluated the system recruiting clinicians with different experience levels in visual tool-based rectal examination.

The study investigated if the visual anorectal examination simulator would prove a valid training tool. Asking participants to perform rectal examination on a variety of conditions, including both cancer and non-cancer, we found that most of them (94%) could identify normal vs. abnormal lesions. Making this identification is the first step in using the tool for diagnosis. Furthermore, the post-questionnaire study results were overwhelmingly positive, with 82% of participants reporting the tool as easy to use and 71% as visually realistic. Overall, 94% of participants agreed that the simulator was useful as a training tool and would recommend it to others.

We have identified a few areas to improve the usability of the system. Replacing the wired magnetic tracking system with a wireless motion sensor can make the system compact and portable. In addition, we have plans to improve the software to include more conditions and various stages of cancer conditions.

## Figures and Tables

**Table 1:** List of conditions virtually recreated in the software program.

Reference Condition	Name	Sigmoidoscope	Proctoscope
RC01	Healthy	X	X
RC02	Anal Fissure		X
RC03	Anal Fistula		X
RC04	Carcinoma	X	X
RC05	Crohn’s disease	X	X
RC06	Diverticulitis	X	
RC07	Haemorrhoids		X
RC08	Polyps	X	X
RC09	Radiation proctitis	X	X
RC10	Ulcer	X	X
RC11	Ulcerative colitis	X	X
RC12	Genital warts		X

**Table 2:** Post-study questionnaire.

Face Validity	Content Validity
1. Tool insertion/withdrawal was realistic	1. The tool was easy to use
2. The operating/manoeuvring of the tool was realistic	2. The range of conditions were broad
3. The tool manipulation was visually realistic	3. The simulator had some hardware/software limitations as a teaching tool
4. The modelled conditions were visually realistic	4. Realism was affected by the modification to the tool
5. The visual observation through the round display looked real	5. How useful is the simulator as a training tool?
6. The size and resolution of the images were adequate	6. How likely are you to recommend the tool to others?
7. The tool movement and displayed images were synchronized	
8. The attachment of the round display and tracking sensor to the tool was not obstructive	

**Table 3:** List of participants.

Group	Number of Participants
Core Surgical Trainees	3
Higher Surgical Trainees	5
Specialist Surgical Registrars	2
Consultant Surgeons	7

**Table 4:** Participant answers regarding how rectal examination training can be improved.

Participant	Answer
1	No exposure prior to training—need realistic exposure
2	Training/teaching sessions
3	More hands-on practice
4	More simulation in medical school training
5	Increased quantity of training
6	Virtual reality simulator would be really helpful
7	Simulation is valuable—need to have high realistic models with accurate feedback
8	More examination/palpation of actual pathology
9	Clinical supervision and training in cases in clinic and in theatre
10	Increased practice with realistic models (due to patients wishes)
11	More exposure to trainees and students
12	Virtual reality

## Data Availability

The datasets presented in this article are not readily available because of ethical restrictions. Requests to access the datasets should be directed to the corresponding author.
